# Balance Assessment in Juvenile Idiopathic Arthritis: A Literature Review

**DOI:** 10.3390/life15040513

**Published:** 2025-03-21

**Authors:** Elena Amaricai, Andrei Daniel Bolovan, Alin Cristian Micuta, Marius Militaru, Anda Gabriela Militaru, Ana Ardelean

**Affiliations:** 1 Department of Rehabilitation, Physical Medicine and Rheumatology, Research Center for Assessment of Human Motion, Functionality and Disability, “Victor Babes” University of Medicine and Pharmacy, 300041 Timisoara, Romania; amaricai.elena@umft.ro; 2 Doctoral School, “Victor Babes” University of Medicine and Pharmacy, 300041 Timisoara, Romania; 3 “Victor Babes” University of Medicine and Pharmacy, 300041 Timisoara, Romania; alin-cristian.micuta@student.umft.ro; 4 Department VIII, Neuroscience, Discipline of Neurology II, Center of Advanced Research in Cardiology and Hemostaseology, “Victor Babes” University of Medicine and Pharmacy, 300041 Timisoara, Romania; marius.militaru@umft.ro; 5 Municipal Emergency Hospital Timisoara, 300254 Timisoara, Romania; militaru.anda@umft.ro; 6 Department V, Internal Medicine I, Discipline of Medical Semiology I, Center of Advanced Research in Cardiology and Hemostaseology, “Victor Babes” University of Medicine and Pharmacy, 300041 Timisoara, Romania; 7 Timisoara Emergency County Hospital, 300723 Timisoara, Romania; ardelean2ana@gmail.com

**Keywords:** juvenile arthritis, postural balance, balance assessment

## Abstract

Juvenile idiopathic arthritis is an inflammatory disease, and children with lower limb involvement have impaired balance compared with healthy peers. The objective of this review was to identify balance instruments used in clinical practice for balance testing in children with juvenile idiopathic arthritis. Three independent reviewers searched the PubMed/Medline, Web of Science, Cochrane, Scopus, and Science Direct databases to identify relevant studies published before 3 March 2025. Five studies were included in the review. Two studies investigated the use of specific tests for balance assessment in children with juvenile idiopathic arthritis (the Bruininks–Oseretsky Test of Motor Proficiency, Second Edition Short Form for motor skills, including balance, the Functional Reach Test for static balance, and the Flamingo Balance Test for postural balance). Three studies used balance testing systems (the S3-Check balance board, the FreeMed posturography system, and the Biodex Balance System). Patients who performed physical exercise programs (including clinical Pilates, strengthening exercises, proprioceptive balance exercises, or home exercises) had significant balance improvements. There are various ways to assess the balance in children suffering from juvenile idiopathic arthritis. None of the review studies used both the specific tests and testing systems. Future research targeting the evaluation of static and dynamic balance through combined tests and equipment is needed. Physical exercise should be an integral part of managing patients suffering from juvenile idiopathic arthritis, as postural control is linked to the overall functioning of this category of patients, who should be involved in recreational activities.

## 1. Introduction

Juvenile idiopathic arthritis (JIA) is an inflammatory disease that can cause joint deformity and destruction, growth abnormalities, and developmental delays. Patients may experience pain, impaired mental health, or difficulty with daily activities [1].

The knee is JIA’s most commonly affected joint, and its early involvement causes quadriceps weakness. Foot deformities include claw toe, valgus or varus hindfoot, and ankle plantar flexion contracture. Arthritis may alter the load on unaccustomed joints [2,3]. The increased sitting time due to painful walking leads to flexion contractures in the lower extremities and muscle atrophy [4]. The arthritis symptoms can lead to muscle weakness around the involved joints, reduced range of motion of the affected joints, impaired aerobic fitness, fatigue, decreased physical activity, and a higher risk of fracture [5,6].

Muscle strength plays a significant role in obtaining an optimal balance. The study by Bozcuk et al. showed that children and adolescents with JIA performed lower than their healthy peers in muscle and cardiovascular capacity tests. Their functional abilities are impaired with higher pain levels and decreased well-being compared with healthy peers [7].

Studies have shown that JIA children with lower limb involvement have an impaired balance ability compared with healthy subjects [8,9]. The study by Yildiz-Kabak et al. pointed out that most children with JIA had impairments in physical functions, activity, and participation [10]. The ability to maintain static and dynamic balance is essential for acquiring motor control and performing the activities of daily life. If balance is impaired during childhood, it can alter the child’s capacity to acquire complex motor skills and practice leisure sports without the risk of injuries [11,12].

The study by Elnaggar et al. showed that the balance-proprioceptive training and strengthening exercises added to conventional therapy in children with polyarticular JIA increased core stability. Moreover, these patients had a higher functional capacity (measured by the 6 min walk test) compared with those who followed conventional therapy only [13].

The balance-proprioceptive exercises, along with weight-bearing tasks, encouraged patients with polyarticular JIA to engage in daily activities, resulting in better functional outcomes measured by the 9 min run–walk test [14]. Our review hypothesis began with the question of whether there is a tailored assessment tool for evaluating balance in children diagnosed with JIA. The disability caused by JIA is extensively studied [15,16].

In contrast, the postural stability resulting from the involvement of the lower limbs is a topic of less interest. However, the consequences of balance impairments and the risk of falls should be clinically significant. Prevention strategies for this group of patients must be included in the overall management of children suffering from JIA.

The objective of our review was to identify balance tools used in clinical practice aimed at assessing balance in children with juvenile idiopathic arthritis.

## 2. Materials and Methods

Three independent reviewers (E.A., A.D.B. and A.A.) performed an Internet-based search of five databases (PubMed/Medline, Web of Science, Cochrane, Scopus, and Science Direct) to identify relevant studies published before 3 March 2025. The keywords were: “assessment” AND “tool” AND “balance” AND “juvenile idiopathic arthritis”; “evaluation” AND “tool” AND “balance” AND “juvenile idiopathic arthritis”; “assessment” AND “tool” AND “postural control” AND “juvenile idiopathic arthritis”; “evaluation” AND “ tool” AND “postural control” AND “juvenile idiopathic arthritis”; “assessment” AND “instrument” AND “balance” AND “juvenile idiopathic arthritis”; “evaluation” AND “instrument” AND “balance” AND “juvenile idiopathic arthritis”; “assessment” AND “instrument” AND “postural control” AND “juvenile idiopathic arthritis”; “evaluation” AND “ instrument” AND “postural control” AND “juvenile idiopathic arthritis.”

We included papers in our review if they described a tool that fulfilled the following criteria: the tool was intended for clinical use, and assessments were conducted by a clinician either in a hospital or the community, regardless of whether laboratory equipment was necessary. Furthermore, the tool’s measurement properties were assessed in individuals over 6 years old with JIA (the age considered for evaluating postural stability) [17].

The exclusion criteria included the following: not full-text articles; reviews; case studies; not published in English language; and not addressing balance assessment.

The three independent reviewers (E.A., A.D.B. and A.A.) performed the selection, inclusion of studies and data extraction.

Three independent reviewers evaluated the studies for bias risk, and any disagreements were resolved with the help of a fourth reviewer (M.M.). The assessment utilized the modified cross-sectional tool (AXIS) [18], which focused on clear objectives, suitable design, well-defined and adequate sample size, measured results, appropriate instruments, clear statistical analysis, significance determination, and a sufficiently reproducible method.

**Figure 1:**
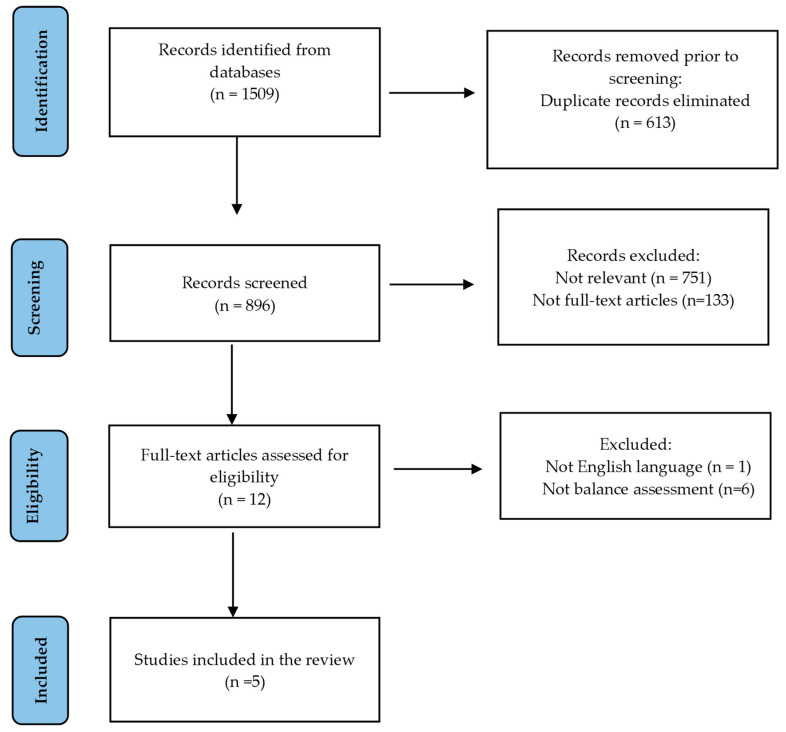
Selection of the included studies.

Figure 1 presents the selection of the studies included in the analysis. Table 1 summarizes the included studies.

All the studies included in the review had clearly stated objectives. The sample size was sufficient, and the target population was represented by JIA patients. All the studies had a clear definition of the condition (JIA), a validated instrument to assess it, and a relevant statistical analysis for the analyzed data. The results were adequately presented. The discussions and conclusions were supported by the results of the studies. Limitations were addressed appropriately.

## 3. Results

### 3.1. Specific Tests Used for Balance Assessment

The study by Calik et al. used the Bruininks–Oseretsky Test of Motor Proficiency Second Edition Short Form (BOT-2 SF) [19]. It assesses motor skills and consists of eight subtests (fine motor precision, fine motor integration, manual dexterity, bilateral coordination, balance, running speed and agility, upper limb coordination, and strength). The balance subtest assesses the motor skills necessary for posture control during activities such as standing, walking, or reaching. A score indicates that individuals can sustain stability while standing on one leg on a balance beam with their eyes open for over 10 s and likely for 5 to 10 s with their eyes closed [23].

Fifteen children diagnosed with JIA were randomly divided into two groups: a clinical Pilates exercise group (focused on respiration, rib cage placement, shoulder placement, and head and neck placement) and a home exercise group (consisting of stretching and strengthening exercises for the whole body). The programs were performed three times per week for 6 weeks. When analyzing the within-group results, significant improvements were recorded in the BOT-2 SF balance subtest for both groups at the post-treatment assessment. However, no significant differences were recorded in the balance subtest when comparing the final outcomes between the clinical Pilates exercise group and the home exercise group [19].

The study by Baydogan et al. analyzed the static balance using the Functional Reach Test (FRT) and postural balance control using the Flamingo Balance Test (FBT) [20]. The FRT was performed with the participant in a standing position. Using a fixed base of support, it measures the difference (in centimeters) between the arm’s length with the arm at 90° flexion and maximal forward reach. The FRT score correlates with the anteroposterior excursion of the center of pressure [24,25,26]. In the FBT, the subject is standing on the preferred foot, bends the free leg backward, and grips the back of the foot with the hand on the same side, standing like a flamingo [27].

Thirty JIA patients were randomly included in group 1 (strengthening exercise group: bicycle ergometer, lower extremity muscle stretching, and strengthening exercises) and group 2 (proprioceptive balance exercise group: bicycle ergometer, lower extremity muscle stretching, and additional proprioceptive-balance exercises). The programs were performed for 12 weeks (36 supervised sessions). After treatment, the intragroup analysis showed statistically significant improvements (*p* ˂ 0.001) in both groups for balance assessment (after treatment differences- FRT: group 1 = 6 cm, group 2 = 12 cm; FBT: group 1 = −2 number of falls, group 2 = −10 number of falls). The intergroup analysis recorded statistically significant improvements in postural balance in the proprioceptive balance exercise group (after treatment differences in FRT: *p* = 0.015, in FBT: *p* = 0.009) [20].

### 3.2. Balance Testing Systems

Merker et al. investigated the postural control on an unstable surface in different groups: active JIA group (swollen and/or painful joints of at least one lower limb joint at the time of testing), inactive JIA group (inactive synovitis of lower extremity joints), and healthy controls [21]. Postural control was measured with the S3-Check (TST, Großhoeflein, Austria). The children were standing barefoot on the S3-Check with their feet shoulder-width apart and their eyes open, and they were told to keep the board as horizontal as possible for 30 s. The following parameters were recorded: sensorimotor index (SMI), symmetry index (SYI), and stability index (STI). The SMI provides information about the sensorimotor ability to regulate during a balancing exercise. The SYI describes deviations of the balance board to the left and right, indicating the ability to keep the board horizontal during testing time. The STI marks the complex sensorimotor performance of the tested subjects. It gives information about the ability of the participants to control their posture and to steady their bodies in a balance task [28].

The adjusted means of SMI and STI were significantly lower in both patient groups compared with controls, indicating better postural stability and sensorimotor regulation ability in patients than in healthy subjects (*p* < 0.001). No differences were observed in SMI (*p* = 0.365) and STI (*p* > 0.999) between the active and inactive JIA groups. The study recorded weak correlations between balance indices and disease-related variables (age of JIA onset, duration of JIA, active joint count in total and the lower extremities, number of joints with limited range of motion, physician and patient global assessment of overall disease activity, pain intensity, history of physiotherapy).

Patti et al. [22] assessed the postural control using the posturography analysis. Nineteen JIA children and 39 healthy controls underwent a posturography test with the FreeMed posturography system (FreeMed baropodometric platform and FreeStep v.1.0.3 software, Sensor Medica, Guidonia Montecelio, Roma, Italy). Additionally, all subjects underwent posturography analysis using the Romberg test position [29]. The balance parameters included the length of the sway path of the center of pressure (CoP), the area of the ellipse, and the coordinates of the CoP along the frontal (X-mean) and sagittal (Y-mean) planes [30].

Posturography data in the JIA group were significantly higher compared with the control group in the length of the sway path of the CoP (JIA group 921.2 ± 430.7 mm vs. control group 543.2 ± 300.2 mm; *p* < 0.001) and ellipse surface area (JIA group 165.8 ± 215.7 mm^2^ vs. control group 84.47 ± 47.94 mm^2^; *p* < 0.05). X and Y mean were not statistically significantly different among the three groups [22].

The study by Houghton et al. assessed the balance in children with lower limb involvement due to juvenile idiopathic arthritis [9]. Twenty-five JIA children with lower extremity arthritis were diagnosed in the past year, and 36 healthy controls were examined by the Biodex Balance System (Biodex, Shirley, NY, USA). The Biodex Balance System features a multiaxial tilting platform designed for precise measurement of an individual’s capacity to maintain dynamic single- and double-limb postural stability on an unstable surface. This adjustable balance platform can tilt up to 20° across a full 360° range of motion. By employing stabilometric techniques, it evaluates both overall (OA) single- and double-limb postural stability, as well as anterior/posterior (AP) and medial/lateral (ML) stability. The OA stability index quantifies the degree of foot platform displacement from level across all movements during a test. The stability index further measures the angular movement of the patient’s center of gravity during static assessments. The AP stability score indicates variations in foot platform displacement from level for movements in the sagittal plane, while the ML stability score reflects foot platform displacement in the frontal plane. A higher score indicates poorer balance. Among these, the OA stability score is the most accurate gauge of the patient’s ability to stabilize the platform [31].

The stability platform accommodates various difficulty levels for stability testing, ranging from level 12 (static) to level 1 (least stable). Average stability scores for OA, AP, and ML were measured under five conditions: right-leg static balance, left-leg static balance, bilateral static balance, bilateral dynamic balance at Biodex Balance System level 2 (very unstable), and bilateral dynamic balance at level 7 (moderately unstable). The authors discovered that children and adolescents with JIA who had a history of lower extremity arthritis within the past year exhibited significant deficits in single-leg balance and mild deficits in bilateral dynamic balance compared with healthy controls. Notably, single-leg balance was considerably impaired, with 40% of children with JIA unable to maintain a one-leg stance on a stable surface for 20 s with their eyes open. Both groups experienced errors and balance loss, but only the control group showed a reduction over trials, indicating a training or learning effect among the healthy participants. The JIA group’s stability indices for AP, ML, and OA at level 2 (very unstable) were significantly higher than those of the controls. No notable differences emerged between the JIA group and healthy controls in bilateral static or dynamic balance at level 7 (moderately unstable). Overall, the study found mild impairments in bilateral dynamic balance at Biodex Balance System level 2 (very unstable) but no impairments in bilateral static balance at level 12 or in dynamic balance at level 7 (moderately unstable) [9].

## 4. Discussion

The aim of the current review was to identify tools for balance testing in children diagnosed with JIA. We found that exercise, be it strengthening, proprioceptive balance [19,20], or Pilates [19], improves symptoms caused by JIA and overall balance in this category of patients [9,21,22].

Baydogan et al. concluded that proprioceptive-balance exercises are better than strengthening when enhancing daily activities such as walking and climbing stairs [20]. This is acquired through improved muscle strength and balance parameters. Clinical Pilates was investigated by Calik et al. and the authors reported improvements in upper limb coordination, daily activities, manual dexterity, and running speed agility in children diagnosed with JIA [19]. Houghton et al. found that, when comparing JIA patients with healthy controls, the first group had a severely affected single-leg balance and a moderately affected bilateral dynamic balance. There were no differences in balance scores between children with painful or painless JIA [9].

Merker and Patti’s findings on postural control contradict each other. The first study states that the JIA group has better postural control than the control group, while the second study found lower values in JIA patients [15,16]. Given that Merker matched the JIA and control group in number, age, and gender, it could mean that this study’s results are more accurate. However, this topic needs to be further investigated.

The study by Leblebici et al. investigated the effects of functional improvement on gait and balance in 18 children with unilateral arm-affected rheumatic diseases (JIA, scleroderma, etc.) [32]. The dynamic balance (single-limb support duration) and overall center of force (CoF) were assessed initially and after a 6-week upper extremity rehabilitation program. Initially, the single-limb support duration was significantly longer, and the displacement of the CoF was considerably lower on the affected side than on the unaffected side. After rehabilitation, the single-limb support duration decreased on the affected side and increased on the unaffected side. Post-treatment, there were no differences in the displacement of the CoF between the affected and unaffected sides (*p* > 0.05). The authors stated that in children with unilateral arm-affected rheumatic diseases (including JIA), upper extremity rehabilitation may increase the dynamic balance by reducing the difference between the affected and unaffected extremities in walking, thus facilitating a more symmetrical weight transfer.

To our knowledge, the current review is the first summary to investigate balance in children with JIA. Similar reviews existing in the literature have assessed the effectiveness of balance training in improving functional capacity in adult patients with rheumatoid arthritis (RA). Brenton-Rule et al. showed that balance was significantly impaired in adults with RA due to muscle weakness and foot deformity, which could represent a fall risk factor for these patients [33]. It was found that dynamic postural stability was affected by functional status and fall risk could be independent of the disease activity. Silva KN et al. concluded that proprioceptive training alone did not have enough evidence to support its application in RA patients, even though it showed improvements in other populations [34].

The small number of included studies is a limitation of our review. All five studies used different tools for balance assessment (specific tests or testing systems); the different reported measures restricted us from performing a meta-analysis. The heterogeneity of the studies represents another limitation. In their review, Sibley et al. synthesized the existing literature on validated balance measures for children, highlighting that pediatric balance assessments often offer a limited analysis of postural control. The theoretical aspects of postural control incorporated in standardized balance measures for children are diverse. Yet, they fail to deliver a thorough evaluation of all essential components of standing posture control [35]. However, the review by Sibley et al. focused on the broader pediatric population, while our review specifically targets children suffering from JIA. This can explain the difference in the number of studies included (21 studies in the review by Sibley et al. versus five studies in the current review).

## 5. Conclusions

Children suffering from JIA have impaired balance compared with their healthy peers. However, JIA patients who have undergone different physical exercise programs experience improved static balance. Future research targeting static and dynamic balance evaluation through combined tests and equipment is needed. Physical exercise should be an integral part of managing JIA patients, as postural control is linked to the overall functioning of this category of patients who should be involved in recreational activities.

## Figures and Tables

**Table 1:** Summary of the included studies.

Authors/ Year of Publication	Study Design	Number of JIA Patients	Number of Healthy Controls	Group Comparison/ Intervention	Balance Assessment	Measures Reported	Outcome Measures
Calik et al. [19]/2020	Randomized controlled trial	15 (mean age: 12.00 ± 3.4 years)	-	Clinical Pilates exercise group (*n* = 6) Home exercise group (*n* = 9) For both groups: 3 times a week, 6 weeks	Bruininks–Oseretsky Test of Motor Proficiency Second Edition Short Form (BOT-2 SF)-Balance subset	-Walking forward on a line (number of steps) - Standing on one leg on a balance beam with eyes open for 10 s	Both groups: no significant differences in balance subset parameters after treatment
Baydogan et al. [20]/2015	Randomized, single-blind, controlled trial	30 Group 1: 15 (mean age: 9.27 ± 1.43 years) Group 2: 15 (mean age: 10.00 ± 3.66 years)	-	Group 1: Strengthening exercises group (*n* = 15) Group 2: Proprioceptive balance exercise group (*n* = 15) For both groups: 36 supervised sessions (12-week period).	Functional Reach Test (FRT) for static balance Flamingo Balance Test for postural balance control	The difference measured (in centimeters) between arm length at 90° flexion and maximum forward reach, with a stable base of support. Number of falls during 1-min stance	FRT after treatment: -statistically significant improvements in both groups (intragroup comparison) -statistically significant improvements in group 2 (intergroup comparison) Postural balance control after treatment: -statistically significant improvements in both groups (intragroup comparison) -statistically significant improvements in group 2 (intergroup comparison)
Merker et al. [21]/2017	Cross- sectional study	72 -Group 1: active JIA (mean age: 13.7 ± 3.4 years) -Group 2: inactive JIA (mean age: 13.5 ± 3.1 years)	36 (mean age: 13.3 ± 3.2 years)	Group 1 Group 2 Control group: age and gender matched healthy children	Postural control measured with the S3-Check (TST, Großhoeflein, Austria)	-Sensorimotor index (SMI) -Symmetry index (SYI) -Stability index (STI)	STI and SMI: no differences between the active and inactive JIA group. SYI: within the normal range (40:60% to 50:50%); none of the groups had any preferences for one side.
Patti et al. [22]/2017	Pilot study	19 (mean age: 12.23 ± 4.46 years)	39 (mean age: 12.87 ± 3.04 years)	JIA patients Healthy controls	Posturography test: FreeMed posturography system (the FreeMed baropodometric platform and FreeStep v.1.0.3 software, Sensor Medica, Guidonia Montecelio, Roma, Italy)	-Length of sway path of the CoP -Ellipse surface area -Coordinates of the CoP coordinates along the frontal (X-mean) and sagittal (Y-mean) planes	Length of the sway path of the CoP and ellipse surface area: significantly higher in JIA group X and Y mean: no statistically significant differences between JIA group and controls.
Houghton et al. [9]/2013	Cross- sectional study	25 (mean age: 13.5 ± 2.5 years)	36 (age range: 8.2–18.0 years)	JIA patients Healthy controls	Biodex Balance System (BBS; Biodex, Shirley, New York)	-Overall (OA) single- and double-limb postural stability -Anterior/Posterior (AP) stability -Medial/Lateral (ML) stability	AP, ML, and OA stability indices (at very unstable level): significantly higher in JIA group than controls. Bilateral static balance and dynamic balance (at moderately unstable level): no significant differences between JIA group and controls.

JIA: juvenile idiopathic arthritis; CoP: center of pressure.

## Data Availability

The data presented in this study are available from the corresponding author (A.D.B.) upon request.

## Abbreviations

The following abbreviations are used in this manuscript: juvenile idiopathic arthritis (JIA), center of pressure (CoP), Functional Reach Test (FRT), Flamingo Balance Test (FBT), sensorimotor index (SMI), symmetry index (SYI), stability index (STI), center of force (CoF), rheumatoid arthritis (RA).
